# Early visual deprivation disrupts the mental representation of numbers in visually impaired children

**DOI:** 10.1038/s41598-022-25044-1

**Published:** 2022-12-29

**Authors:** G. Cappagli, L. F. Cuturi, S. Signorini, F. Morelli, E. Cocchi, M. Gori

**Affiliations:** 1 Unit for Visually Impaired People (UVIP), Istituto Italiano di Tecnologia, Via Melen 83, 16100 Genova, Italy; 2 Developmental Neuro-Ophthalmology Unit, IRCCS Mondino Foundation, Pavia, Italy; 3 Department of Cognitive, Psychological, Pedagogical Sciences and of Cultural Studies, University of Messina, Messina, Italy; 4 Chiossone Onlus, Genova, Italy; 5 Department of Brain and Behavioural Sciences, University of Pavia, Pavia, Italy

**Keywords:** Psychology, Human behaviour

## Abstract

Several shreds of evidence indicate that visual deprivation does not alter numerical competence neither in adults nor in children. However, studies reporting non-impaired numerical abilities in the visually impaired population present some limitations: (a) they mainly assessed the ability to process numbers (e.g. mathematical competence) rather than represent numbers (e.g. mental number line); (b) they principally focused on positive rather than negative number estimates; (c) they investigated numerical abilities in adult individuals except one focusing on children (Crollen et al. in Cognition 210:104586, 2021). Overall, this could limit a comprehensive explanation of the role exerted by vision on numerical processing when vision is compromised. Here we investigated how congenital visual deprivation affects the ability to represent positive and negative numbers in horizontal and sagittal planes in visually impaired children (thirteen children with low vision, eight children with complete blindness, age range 6–15 years old). We adapted the number-to-position paradigm adopted by Crollen et al. (Cognition 210:104586, 2021), asking children to indicate the spatial position of positive and negative numbers on a graduated rule positioned horizontally or sagittally in the frontal plane. Results suggest that long-term visual deprivation alters the ability to identify the spatial position of numbers independently of the spatial plane and the number polarity. Moreover, results indicate that relying on poor visual acuity is detrimental for low vision children when asked to localize both positive and negative numbers in space, suggesting that visual experience might have a differential role in numerical processing depending on number polarity. Such findings add knowledge related to the impact of visual experience on numerical processing. Since both positive and negative numbers are fundamental aspects of learning mathematical principles, the outcomes of the present study inform about the need to implement early rehabilitation strategies to prevent the risk of numerical difficulties in visually impaired children.

## Introduction

Vision is deemed fundamental for numerical perception and several evidence indicates that it shapes the development of basic numerical skills^1^, mainly because vision is the leading sense in spatial cognition and numbers are systematically associated with space^2–7^. Indeed, vision senses numerosity directly without the involvement of cognitive strategies and the fact that numerosity is strongly susceptible to visual adaptation suggests that numerosity is a primary visual property^8,9^. Therefore, we might expect to observe impaired spatial performance and altered numerical processing and representation in individuals with visual disabilities. Regarding spatial abilities in the visually impaired population, research has shown that visual deprivation from an early age can delay or alter perceptual development^10–15^. For instance, it has been recently shown that visual experience is essential for flexible spatial non-visual perspective-taking. Indeed, blindness reduces the adoption of decentered perspectives in the tactile domain^16^, and low vision hampers the ability to switch from egocentric to allocentric visual perspectives^17^. Similarly, in the auditory domain, visually impaired individuals might present a bias in perceiving the spatial location of a sound^18^ and lower accuracy in detecting the spatial distance of sound sources in the environment^19^. Studies concerning numerical abilities agree about the non-detrimental role of visual loss or deprivation on numerical processing^20^. For instance, empirical evidence has demonstrated that early blind individuals are proficient in various numerical tasks^20,21^. Early blind adults present unaltered numerosity estimation and comparison^22–25^, counting^26^ and calculation skills^27^. Moreover, visually impaired adults tend to spontaneously represent numbers in a spatial format on a mental number line, similar to sighted individuals^11^, presenting a normal semantic representation of numbers^28^. Finally, blind individuals show sighted-like bisection and pseudoneglect effects in several numerical tasks^29–32^. Similarly in the geometrical domain, studies indicate that blind children^33^ and adults^33,34^ present the same profile of difficulties than sighted controls when asked to haptically recognize a deviant stimulus among similar shapes, but also a lower performance when shapes were visually presented. Overall, such studies support the hypothesis that visual experience and visual feedback are not necessary for numerical processing abilities, which can be acquired through touch, audition and higher cognitive functions such as language^27,35^ despite some evidences indicate that vision influences the development of specific numerical competencies such as finger–number interactions^20,26^. However, studies investigating numerical abilities in the visually impaired population present some limitations. Firstly, they mainly assessed numerical performance intended as the ability to process numbers (e.g., mathematical operations), which requires a certain degree of cognitive effort, rather than represent numbers in space (e.g. mental number line), which are thought to be influenced by visuospatial abilities. For instance, visually impaired individuals were asked to count the number of dissimilar syllables in a sequence presented auditorily^36^ or count different auditory stimuli^36^, perform mathematical operations or bisect numerical intervals given through audition^27,29–31,37^. Such tasks could potentially limit a broader understanding of how numerical competence develops in the blind, because they are at least partially grounded on cognitive strategies, possibly involving working memory competencies. Secondly, existing studies principally investigated positive rather than negative number estimation and assessed numerical performance on horizontal rather than other spatial planes. Such knowledge would be fundamental at the scientific level in order to understand whether visual deprivation affects the ability to represent numbers and at the clinical level in order to design the best rehabilitation practices to support numerical competence in visually impaired individuals. Indeed, it is well known that blind individuals represent numerical information in the form of a mental number line as they show the typical spatial-numerical association of response codes and the same overestimation of the left side of the mental number line as sighted individuals do (for a review, see^21^). Nonetheless, to our knowledge, no published studies investigated how the lack of visual experience alters or prevents the representation of numerical information across different planes (i.e., sagittal). Finally, most of existing studies investigated numerical performance in visually impaired adults rather than children. To our knowledge, only one study^38^ assessed the performance of congenitally blind children in a series of visuospatial numerical tasks (number-to-position, number bisection, counting, and mathematical operations). The authors reported that visual deprivation from birth does not affect numerical performance, which seems to affect the association between arithmetic competence and working memory mechanisms. Nonetheless, numerical performance was principally investigated with auditory tasks based on working memory capabilities (number bisection, counting and mathematical operations), as in a previous paper examining the spontaneous use of finger-counting strategies as a way to develop basic numerical abilities^36^. Moreover, the only haptic task based on number representation was administered on the horizontal plane with positive numbers. Understanding whether visually impaired individuals may develop numerical impairments according to their visual residuals and when such impairments may manifest during growth would be of fundamental importance for therapeutic purposes, since rehabilitation protocols might be specifically targeted on the perceptual deficits spotted from early infancy. To fill this gap, we investigated how visual deprivation from birth impacts number representation by administering a revised version of the tactile number-to-position task proposed by Crollen et al.^38^ to low vision and blind children, requiring them to position positive and negative numbers on the horizontal and the sagittal planes. We hypothesize that visually impaired children would show a worsen performance when asked to represent numbers in the sagittal instead of the horizontal plane. Indeed it has been demonstrated that visual experience might differently affect the calibration of spatial dimensions such as horizontal and vertical axes^39,40^. Moreover, we hypothesize that number polarity (positive vs. negative) would not significantly affect visually impaired children’s performance, because negative numbers are associated with full-body movements going backward^41^ and studies have shown that blind individuals are not impaired in spatial localization in the rear space^42,43^.

## Material and methods

### Task and procedure

**Figure 1:**
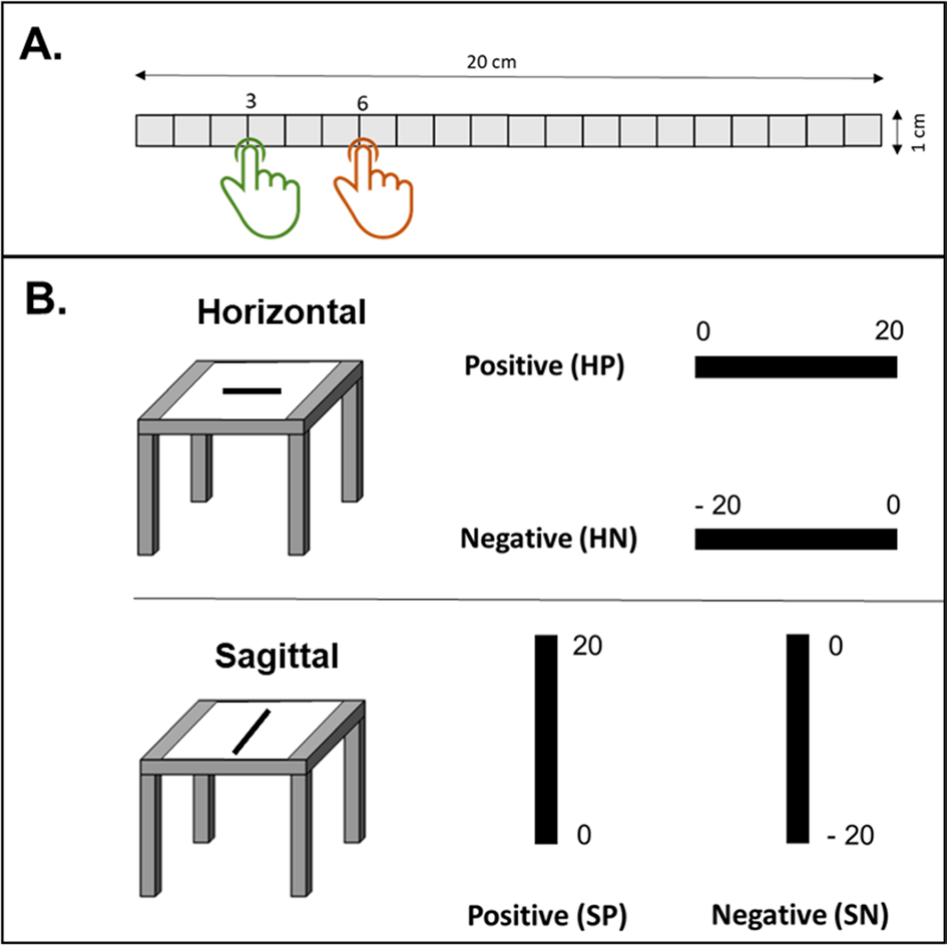
(**A**) Haptic number-to-position task. On each trial, children were required to localize numbers on a metal rule, and deviation from true numbers’ position was calculated in terms of PE (percentage of error) and PAE (percentage of absolute error). (**B**) Task conditions. The task comprised four task conditions depending on rule position on the table (horizontal or sagittal) and on number polarity (positive or negative). HP: horizontal number line with positive numbers increasing from left to right; HN: horizontal number line with negative numbers decreasing from left to right; SP: sagittal number line with positive numbers increasing from bottom to top; SN: sagittal number line with negative numbers decreasing from bottom to top.

A haptic number-to-position task was designed with a 20 cm graduated rule embedded in a solid sheet of graph paper positioned on a paper board (Fig. 1A). On each trial, children were required to show where different numbers would be placed on the rule by moving a cursor to the estimated location. Four conditions of the task were randomly administered to each participant (Fig. 1B) by manipulating two variables, namely number polarity (positive vs. negative) and number line orientation (horizontal vs. sagittal): (a) horizontal number line with positive numbers on the rule verbally labeled “0” at its left end and “20” at its right end; (b) horizontal number line with negative numbers on the rule verbally labeled “− 20” at its left end and “0” at its right end; (c) sagittal number line with positive numbers on the rule verbally labeled “0” at its bottom end and “20” at its top end; (d) sagittal number line with negative numbers on the rule verbally labeled “− 20” at its bottom end and “0” at its top end. On each trial, a number was auditorily presented among eleven target numbers from 0 to 20 (3, 4, 6, 8, 10, 12, 14, 16, 17, 18, 19) and children were required to indicate the position of the selected number starting their finger movement from a starting point on the haptic line corresponding to the 0 value. No time restrictions were given to participants in order to accomplish the task. Each participant performed 44 trials, i.e., 11 trials for each condition defined by the spatial plane considered (horizontal vs. sagittal) and the positivity of numbers (positive vs. negative).

Participants were blindfolded in case of visual residual or normal vision. Responses were recorded by the experimenter standing behind the participants’ seats and seeing the graduations of the graph paper. As in Crollen et al.^38^, performance at the task was measured as Percentage of Absolute Error (i.e., PAE), which corresponds to the absolute value of the Percentage of Error (i.e., PE). The PE for each number was computed: ((participant’s number estimation − true number)/line’s scale) * 100. Negative values indicated a left/down bias, and positive values showed a right/up bias. Based on Crollen et al.^38^, the power analysis conducted with G * Power 3^44^, with a medium effect size (d = 0.60) based on meta-analytic reporting of implicit d-SNA tasks^7^ indicated that the sample size recruited for this study was adequate in terms of power (1 − β > 0.90) to detect numerical abilities among the three groups enrolled.

### Participants

Eight congenitally blind (CB) children (3 males; mean age 11 years old; all right-handed), thirteen low vision (LV) children (7 males, mean age 9.9 years old, all right-handed) and twelve sighted (S) controls (9 males, mean age 9.5 years old, two left-handed) participated in the study (N = 32). No statistical difference was found among groups that concerned chronological age (*p* > 0.05). All visually impaired children enrolled in the CB and LV groups were born with visual impairment, did not have any other sensory or cognitive disability, and presented different causes of blindness (see Table 1 for a clinical description of participants). Visually impaired children were recruited from two rehabilitation centers, i.e., the Chiossone Institute (Genova, Italy) and the Developmental Neuro-ophthalmology Unit of the IRCSS Mondino Foundation (Pavia, Italy). All sighted children were recruited from local schools (Genova, Italy). As a measure of general intelligence, the IQ of the visually impaired groups (CB, LV) was estimated by the Verbal Comprehension Index (VCI) of The Wechsler Intelligence Scale for Children-IV and by the mean of the normalized scores obtained at the verbal subtests of the same scale (Similarity, Vocabulary and Comprehension). Visually impaired children reported normal scores both in terms of VCI (M ± SE = 108 ± 5.18; cutoffs = 80–120) and averaged normalized scores at the verbal subtests (M ± SE = 10.23 ± 1.22; cutoffs for each subscale = 7–13). All test procedures were approved by the research ethics board of the IRCSS Mondino Foundation (Pavia, Italy) and by the Ethical Committee of ASL 3 (Genova, Italy). Written informed parental consent was obtained for all the children. Children were tested once, with each session lasting approximately half an hour.

**Table 1:** Demographical and clinical details of the visually impaired groups.

Visual perception	Age	Gender	Visual acuity (LogMAR)	Cause of visual impairment
Blind	12	M	2	Anophthalmia
Low vision	10	F	0.8	Methylmalonic acidemia
Low vision	10	F	1.1	Microphthalmia and Iris coloboma
Blind	8	M	2	Leber Congenital Amaurosis
Low vision	9	M	0.8	Nystagmus and retinal anomalies
Low vision	9	F	0.8	Aniridia
Blind	15	M	2	Retinal dystrophy
Low vision	13	M	0.7	Retinopathy of prematurity
Blind	6	F	2	Retinal dystrophy
Low vision	14	M	0.8	Septo-optic dysplasia
Blind	8	F	2	Leber Congenital Amaurosis
Blind	14	F	2	Leber Congenital Amaurosis
Blind	12	F	2	Retinopathy of prematurity
Blind	14	F	2	Retinopathy of prematurity
Low vision	11	M	1	Oculo-cutaneous albinism
Low vision	8	M	1.3	Iris coloboma
Low vision	7	F	1.69	Microphthalmia (left) and Anophthalmia (right)
Low vision	14	F	1	Pilocytic astrocytoma
Low vision	7	F	1.2	Nystagmus and Retinopathy
Low vision	11	M	0.69	Bilateral optic atrophy
Low vision	6	M	1.2	Bilateral congenital glaucoma

Participants in the visually impaired groups were either congenitally blind (blind group) or congenitally affected by peripheral disabilities causing impoverished visual acuity (low vision group). Visual acuity is reported as LogMAR with values ranging from 0 (normal vision) to complete blindness (2). All participants reported a visual impairment from birth. F, female; M, male.

### Ethics approval

The study was conducted according to the guidelines of the Declaration of Helsinki and approved by the Ethics Committee of Regione Liguria (Ethical Committee of ASL 3, Genova, Italy, Protocol IIT_UVIP_COMP_2019 N. 02/2020, 4 July 2020) and the Ethics Committee of Regione Lombardia (Fondazione IRCCS Policlinico San Matteo, Pavia, Italy, Protocol p-20190061836).

## Results

**Figure 2:**
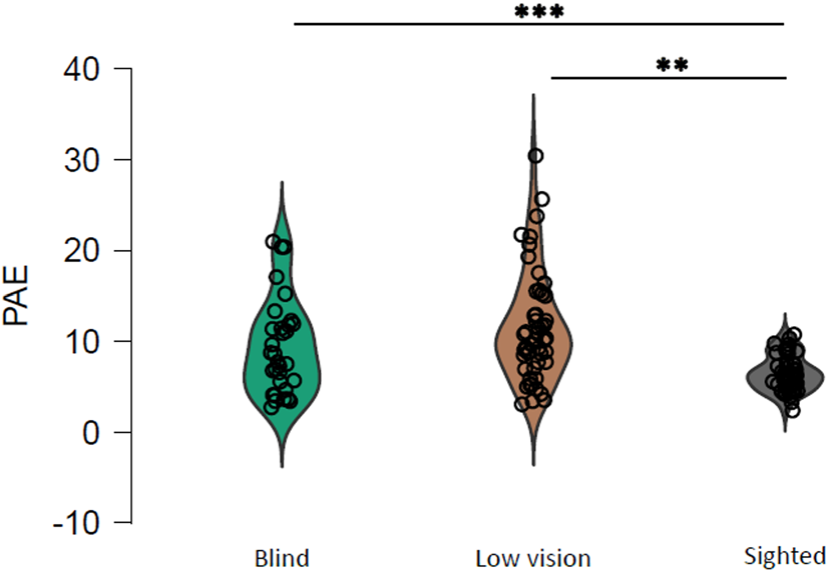
Main effect of visual experience on number-to-position unsigned error. The performance of blindfolded sighted grey), low vision (brown), and congenitally blind (green) children was analyzed in terms of the Percentage of Absolute Error (PAE) representing the absolute deviation to the true number’s position. The graph indicates that visual deprivation significantly alters the ability of children to represent numbers on a tactile rod. The violin plot element shows the density and distribution of the data. Data points show mean data for each participant for each measure. Significant comparisons are highlighted by asterisks (**p < .01; ***p < .001).

Similar to Crollen et al.^38^, we examined both children’s signed bias (PE) and children’s unsigned error (PAE) in the number-to-position task. We used the lme4 package in R to separately fit linear mixed-effects models to raw data after outlier exclusion (threshold at 2 standard deviations from the mean) of PE and PAE separately. For each measure, we then performed a 3 × 2 × 2 ANCOVA on the linear mixed models with group (C for the sighted controls, B for the blind group, and L for the low vision group) as the between-subject factor and polarity (P for positive, N for negative) and orientation (H for horizontal, S for sagittal) as the within-subject factors and age as a covariate. For post-hoc comparison, we performed two-sided, two-sample permutation tests via the permTS function in R’s perm package^45^^,^^46^. Such nonparametric test runs iterations of randomly shuffled data (we use here the mean data for each measure) while maintaining the original sample size of each group. Finally, it calculates the difference in mean for each comparison and returns the *p* value indicating the significance level of the difference between the groups. We report *p* values after Bonferroni correction. For the signed error PE, we found overall no influence of age (F (1, 27. 87) = 0.034, *p* = 0.854). The ANCOVA indicates a significant main effect of vision (F (1, 28.24) = 4.17, *p* = 0.025) and a main effect of polarity (F (1, 1299.87) = 78.67, *p* < 0.001). For vision, post hoc comparisons indicate a trend for a significant difference between blind and low vision children (t = 1.90, *p* = 0.055) but no significant differences between low vision and sighted children (t = 2.35, *p* = 0.170) or blind and sighted children (t = − 0.36, *p* = 1). A one sample permutation t-test does show significant signed errors for sighted (M ± SE = − 0.6 ± 0.5, *p* = 0.237) and blind participants (M ± SE = − 1.1 ± 1.4, *p* = 0.441) and a significant overestimation for low vision (M ± SE = 2.1 ± 0.9, *p* = 0.035). Regarding polarity, all children significantly overestimate when asked to represent negative numbers (M ± SE = 3.35 ± 0.7, *p* < 0.001) while underestimate when asked to represent positive numbers (± SE = − 2.6 ± 0.6, *p* < 0.001) as also shown by a significant difference between positive and negative numbers (t = − 5.45, *p* < 0.001). Overall, the results indicate that total loss of vision from birth (blindness) leads to a more pronounced number underestimation compared to typical and degraded visual conditions. For the unsigned error (PAE), the ANCOVA returns no significant effect of age (F (1, 28.67) = 3.38, *p* = 0.076) but a significant main effect of vision (F (2, 28.74) = 6.28, *p* < 0.01) and post-hoc comparisons suggest that low vision and blind children perform lower at the task compared to sighted children (low vision vs. sighted, t = − 4.81, *p* < 0.001; blind vs. sighted: t = 3.18, *p* < 0.01) whilst we observe no significant differences between blind and low vision children (t = 1.51, *p* = 0.279). Figure 2 represents the unsigned error in each vision group and highlights the detrimental role of visual deprivation on number representation (sighted: M ± SE = 6.5 ± 0.3; low vision: M ± SE = 1.5 ± 0.8; blind: M ± SE = 9.3 ± 0.9).

**Figure 3:**
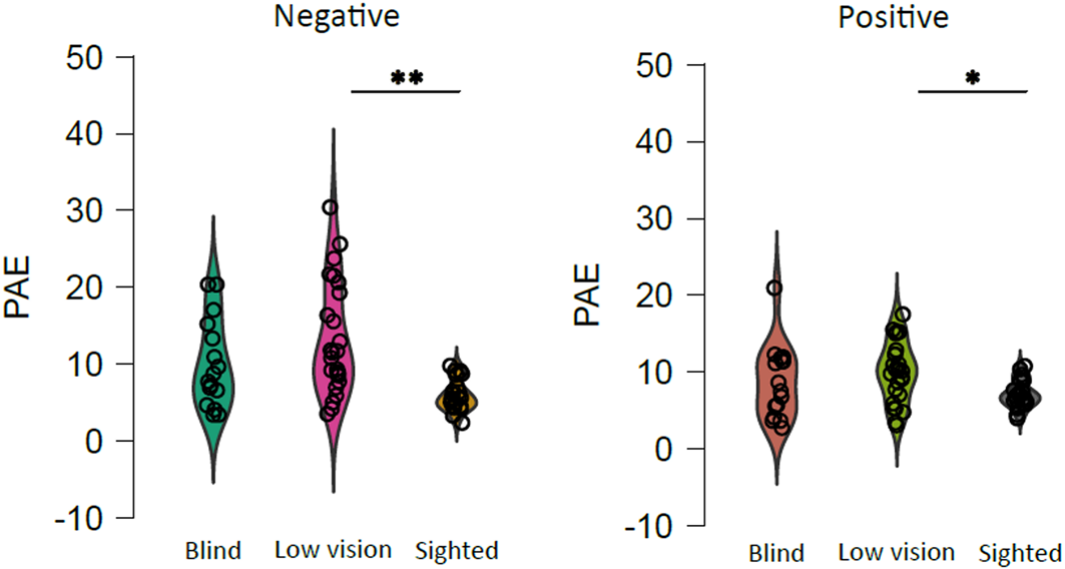
Effect of visual experience and task condition on number-to-position unsigned error. The performance of blindfolded sighted (white), low vision (grey), and congenitally blind (black) children was analyzed in terms of the Percentage of Absolute Error (PAE) representing the absolute deviation to the true number’s position. The graphs indicate that the low vision group differed from the sighted group when asked to localize positive and negative numbers on the rule independently of the spatial plane (horizontal or vertical) considered. The violin plot element shows the density and distribution of the data. Data points represent mean data for each participant. Significant comparisons are highlighted by asterisks (*p < .05; **p < .01).

We observe no significant main effect of polarity (F (1, 1308.89) = 3.08, *p* = 0.079) but a significant interaction between visual impairment and polarity (F (2, 1309.09) = 6.06, *p* < 0.01). Results indicate no significant differences among groups for positive numbers localization (low vision vs. blind: t = 0.91, *p* = 1; blind vs. sighted: t = 1.47, p = 1) except for a significant difference between low vision and sighted (t = 2.94, *p* = 0.04). Also, for negative numbers localization we observe a significant difference between low vison and sighted participants (t = 3.88, *p* < 0.01) but no significant differences among visually impaired groups (low vision vs. blind: t = 1.44, *p* = 1) nor between blind and sighted children (t = 2.85, *p* = 0.064). Figure 3 represents the interaction between visual experience and polarity, with numbers localization being significantly poorer for low vision children independently of the spatial plane considered for both positive (low vision M ± SE: 9.9 ± 0.7; blind M ± SE: 8.6 ± 1.1; sighted M ± SE: 7.1 ± 0.3) and negative numbers (low vision M ± SE: 13.1 ± 1.4; blind M ± SE: 9.9 ± 1.4; sighted M ± SE: 5.8 ± 0.4). Finally, no significant effect of orientation was found (F (1, 1309.94) = 0.77, *p* = 0.377) suggesting that the way numbers are oriented in space (horizontal or sagittal planes) does not influence children’ ability to represent them.

We computed an explorative correlation analysis to investigate whether number localization unsigned error (PAE) is correlated with visual impairment in low vision and blind children as expressed in LogMAR values (from 0 = normal vision to 2 = complete blindness). A negative correlation (R = − 0.20, *p* = 0.196), though non-significant, may suggest that the more the visual impairment the less the absolute error at the task. This result is in agreement with data from the blind children performing even better than low vision children in the negative condition of the task, implying that relying on the visual residual in this task might produce a detrimental effect on the performance.

## Discussion and conclusions

**Figure 4:**
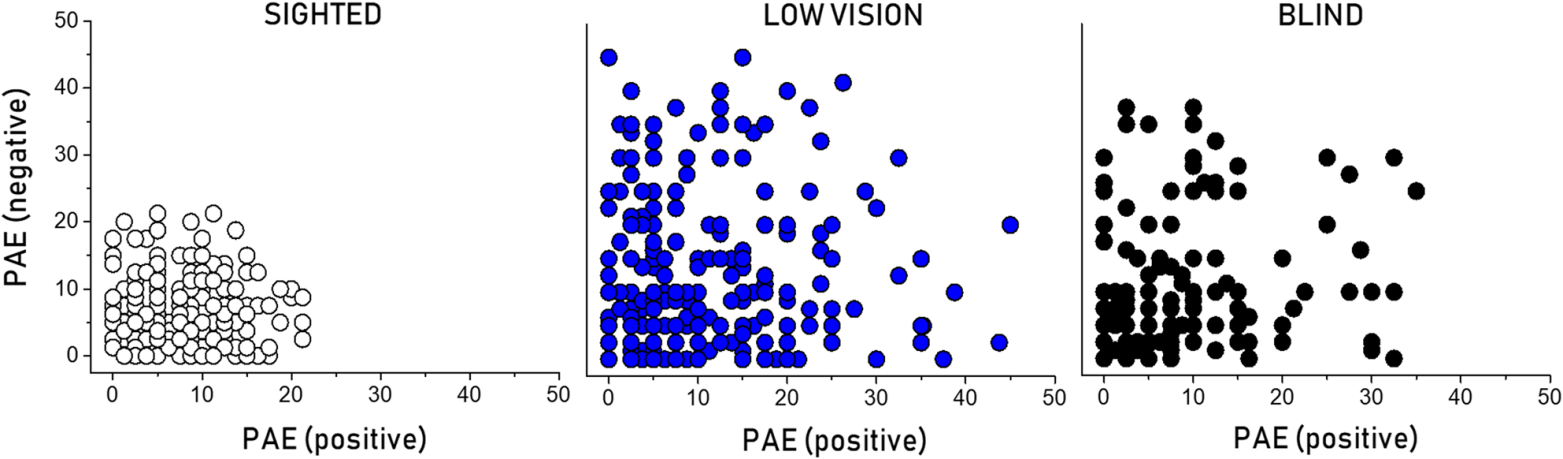
Detrimental effect of visual impairment on negative numbers representation. Individual data of blindfolded sighted (white), low vision (blue) and blind (black) children is graphically represented as Percentage of Absolute Error (PAE) for the positive (x-axis) and negative (y-axis) conditions of the task. Within each plot, the more the distribution shifts to the right the higher the error when representing positive numbers, while the more the distribution shifts to the top the higher the error when representing negative numbers. As can be seen, low vision's performance is particularly shifted to the top and right, indicating lower performance for both positive and negative numbers representation.

Several shreds of evidence indicate that visual deprivation does not alter numerical competence, but most of them only focused on specific spatial-numerical associations (mostly horizontal) and on a specific number polarity (mostly positive). Nonetheless, several studies demonstrate that multiple configurations of spatial-numerical associations might coexist in the horizontal (i.e., left-to-right), vertical (i.e., down-to-up) and sagittal (i.e., near-to-far) space dimensions^47^. The present results indicate that visual deprivation has a detrimental role on number representation independently of how numbers are represented in space (i.e. number orientation) but it specifically alters both positive and negative numbers representation only when visual deprivation is intended as degraded vision from birth, namely low vision. Indeed both blind and low vision children performed worse than sighted peers at the task, but only low vision children performed worse than sighted children for both positive and negative number representation. Studies on typical individuals reveal that numerical processing is grounded on visuospatial mechanisms. Specifically, vision is the most accurate sense to convey spatial information and number representation in space requires vision, thus numbers are systematically associated with space^2,7^. We might expect to find an impaired representation of numbers in individuals affected by different forms of visual deprivation, from partial (low vision) to total (blindness) absence of vision. Nonetheless, several studies assessing numerical representation mainly in terms of mathematical performance (e.g., counting) indicate that visual deprivation does not alter the ability to process or represent numbers neither in adults (e.g.^20,21^) nor in children^38^. Here we found that visual deprivation impairs the ability to represent (and not process) numbers, specifically when visual residual is present but limited (Fig. 4).

The results of this work are in accordance with previous studies showing that the blind population manifest an unaltered numerical performance declined as the ability to represent positive numbers in space and generate mental representations in an “analog” format^28,38^^48^^,^^49^. Our findings might be interpreted as evidence that visual experience does not impact the development of multiple and independent mental number lines, at least for horizontal and sagittal axes^50^. Contrary to our hypotheses, we observed that low vision children present a specific deficit in localizing positive and negative numbers, thus suggesting that number polarity might be a crucial factor in understanding the role of visual experience on numbers representation in space. Likely, visual feedback might be necessary to mentally rotate number position in space. Indeed, when asked to localize negative numbers’ position on the rule in our task, children are implicitly required to build up a specular representation of positive numbers since positive numbers range from 0 to 20 according to a left-to-right orientation and negative numbers go from 0 to − 20 according to a right-to-left direction (example is given for the horizontal condition of the task). It is well known that visual processing facilitates mental rotation of tactile stimuli and visually impaired people demonstrate a typical decrease in performance with increasing stimuli rotation^51,52^. Therefore we might hypothesize that visual deprivation also affects the ability to mentally reverse number polarity.

## Data availability

The datasets generated and analysed during the current study are not publicly available due to privacy restrictions but are available from the corresponding author on reasonable request.
